# 4D trajectory lightweight prediction algorithm based on knowledge distillation technique

**DOI:** 10.3389/fnbot.2025.1643919

**Published:** 2025-08-22

**Authors:** Weizhen Tang, Jie Dai, Zhousheng Huang, Boyang Hao, Weizheng Xie

**Affiliations:** ^1^Civil Aviation Ombudsman Training College, Civil Aviation Flight University of China, Guanghan, China; ^2^College of Air Traffic Management, Civil Aviation Flight University of China, Chengdu, China

**Keywords:** 4D trajectory prediction, multi-step prediction, knowledge distillation technique, Teacher-Student Model, feature extraction

## Abstract

**Introduction:**

To address the challenges of current 4D trajectory prediction—specifically, limited multi-factor feature extraction and excessive computational cost—this study develops a lightweight prediction framework tailored for real-time air-traffic management.

**Methods:**

We propose a hybrid RCBAM–TCN–LSTM architecture enhanced with a teacher–student knowledge distillation mechanism. The Residual Convolutional Block Attention Module (RCBAM) serves as the teacher network to extract high-dimensional spatial features via residual structures and channel–spatial attention. The student network adopts a Temporal Convolutional Network–LSTM (TCN–LSTM) design, integrating dilated causal convolutions and two LSTM layers for efficient temporal modeling. Historical ADS-B trajectory data from Zhuhai Jinwan Airport are preprocessed using cubic spline interpolation and a uniform-step sliding window to ensure data alignment and temporal consistency. In the distillation process, soft labels from the teacher and hard labels from actual observations jointly guide student training

**Results:**

In multi-step prediction experiments, the distilled RCBAM–TCN–LSTM model achieved average reductions of 40%–60% in MAE, RMSE, and MAPE compared with the original RCBAM and TCN–LSTM models, while improving *R*^²^ by 4%–6%. The approach maintained high accuracy across different prediction horizons while reducing computational complexity.

**Discussion:**

The proposed method effectively balances high-precision modeling of spatiotemporal dependencies with lightweight deployment requirements, enabling real-time air-traffic monitoring and early warning on standard CPUs and embedded devices. This framework offers a scalable solution for enhancing the operational safety and efficiency of modern air-traffic control systems.

## 1 Introduction

In recent years, the rapid expansion of the air-transport industry has driven a continuous increase in air-traffic density, leading to frequent flight delays and increasingly congested airspace (Ma et al., 2024). To address the mounting challenges imposed on Air Traffic Control (ATC), the International Civil Aviation Organization (ICAO) has incorporated Trajectory-Based Operations (TBO) into the global aviation framework (Ramasamy et al., 2014). TBO relies on four-dimensional (4D) trajectories—comprising three spatial coordinates plus time—shared dynamically among ATC, airports, airlines, and aircraft to enable collaborative, real-time decision making (Zeng et al., 2022; Hao et al., 2018). In March 2020, the Air Traffic Management Bureau of the Civil Aviation Administration of China (CAAC) published its “Civil Aviation Modernization Strategy (CAAMS) Roadmap,” designating TBO and 4D trajectory prediction as core objectives for future ATC modernization (Vaugeois, 2018; Chang et al., 2020). High-precision trajectory forecasting is therefore essential to enhancing both the safety and efficiency of air-traffic management. Unlike single-step forecasts, multi-step prediction projects an aircraft’s position over an extended horizon, facilitating proactive rerouting and conflict avoidance. However, multi-step trajectory prediction remains subject to severe challenges—cumulative prediction errors, insufficient capture of spatiotemporal dependencies, and high computational costs—which underscore the urgent need to improve its accuracy and efficiency in current aviation research.

Methods for short-term trajectory prediction broadly fall into three categories: kinematic-particle models, state-estimation approaches, and machine-learning techniques. Kinematic-particle models (Peng et al., 2005) treat the aircraft as a point mass, applying force analyses and combining flight-dynamics equations with aircraft parameters to predict motion; however, they demand extensive vehicle-specific and kinematic data. State-estimation methods (Tang et al., 2020) regard aircraft movement as a state-transition process, constructing transition matrices from equations of motion to relate future positions to past states (position, velocity, acceleration). While mathematically rigorous, these linear models struggle with the nonlinear dynamics and external disturbances inherent in real trajectories and incur high computational cost.

Machine learning-based algorithms have gradually been applied to trajectory prediction. Machine learning mines hidden information from large amounts of trajectory data, constructs neural networks, and extracts nonlinear relationships between trajectory information, which is crucial for improving the accuracy of trajectory prediction. Short-term trajectory prediction is divided into single-step prediction and multi-step prediction according to the prediction range. Currently, classical neural networks used for trajectory prediction include Back Propagation (BP) neural networks (Wu et al., 2019), Long Short-Term Memory (LSTM) networks (Shi et al., 2018; Sahadevan et al., 2022; Shi et al., 2019), Gated Recurrent Units (GRU) (Han et al., 2021; Han et al., 2023), and other variants of Recurrent Neural Networks (RNN). Lu et al. (2024) proposed an airport terminal area trajectory prediction model based on a sequence-to-sequence framework, which combines an attention mechanism and an exponential decay sampling method in teacher forcing to predict trajectory sequences, thereby improving prediction accuracy and model training efficiency. Huang and Ding (2022) proposed a novel trajectory prediction model based on Temporal Convolutional Networks (TCN) and Bidirectional Gated Recurrent Units (BiGRU), and optimized the model’s hyperparameters using a Bayesian algorithm. Shi et al. (2024) proposed a multi-step 4D trajectory prediction model (GTA-Seq2Seq) that uses GRU, TCN, and Temporal Pattern Attention (TPA) mechanisms in the sequence-to-sequence (Seq2Seq) model. Experiments showed that the number of parameters required by this model during training was reduced by 67.42% compared with STED (the optimal model), improving the model’s operational efficiency. Shafienya and Regan (2022) proposed a hybrid deep learning method for trajectory prediction at Hartsfield-Jackson International Airport. They preprocessed ADS-B data, fused CNN-GRU with 3D-CNN, enhanced robustness through Monte Carlo dropout, and reduced the post-average error rate by 21%, significantly optimizing prediction errors. Chuan et al. (2023) developed a new model fusing temporal convolutional recurrent neural networks and LSTM networks to enhance the ability to predict future flight routes of aircraft. The model uses the former to capture key navigation attributes and the latter to correct prediction deviations and suppress error accumulation. Simulation experiments verified that the model exhibits high accuracy in route prediction. Dong et al. (2023) proposed a trajectory prediction model that performs deep learning after feature extraction. This hybrid model combines a Temporal Convolutional Network and an improved transformer model, and the results showed that the proposed TCN-Informer architecture performs better in various evaluation metrics. Zhang and Liu (2025) proposed a 4D trajectory prediction method based on K-medoids clustering and Conditional Tabular Generative Adversarial Networks (CTGAN). Comparative experiments with four LSTM-based models and the original CTGAN model demonstrated that the proposed model has significantly higher trajectory prediction accuracy than other models when predicting medium and long-term trajectories. Wu et al. (2022) proposed a long-term 4D trajectory prediction model based on Generative Adversarial Networks (GAN). They designed three deep generative models for trajectory prediction based on 1D Convolutional Neural Networks (Conv1D-GAN), 2D Convolutional Neural Networks (Conv2D-GAN), and LSTM-GAN, and the results indicated that Conv1D-GAN is the most suitable generative adversarial network for long-term aircraft trajectory prediction. LSTM is widely used in processing and predicting time series data. Liu and Hansen (2018) developed an encoder-decoder-based LSTM generative model for predicting aircraft 4D trajectories. Zhao et al. (2019) proposed a Deep LSTM (D-LSTM) neural network to improve the prediction accuracy of aircraft in complex flight environments. Lei et al. (2025) introduced a novel Deep Multimodal Network (DMN), which integrates a shared feature extractor and a multi-task prediction module with a translation encoder to capture intra-modal and inter-modal dependencies. Compared with baseline models using real datasets, it shows superior performance. Li et al. (2025) proposed a noise-robust autoregressive transformer that enhances prediction reliability by integrating noise-regularization embeddings into multi-head attention equipped with hybrid positional encodings. The model effectively captures essential spatiotemporal relationships and more accurately manages positional information across diverse trajectories. Guo et al. (2024) introduced a speech-instruction-aware trajectory-prediction framework that treats controller voice as an independent modality for 4-D trajectory forecasting. By employing a three-stage progressive multimodal learning paradigm, the framework bridges the heterogeneous gap between speech and trajectory data, achieving over 20% relative reduction in mean deviation error on real-world datasets and enabling the first real-time coupling of “control intent–trajectory.” This work opens a new avenue for future 4-D trajectory research by integrating spoken intentions. Gao et al. (2025) proposed the CORR-CNN-BiLSTM-Attention model, which combines convolutional feature extraction, bidirectional LSTM temporal modeling, and attention correction. Applied to projectile trajectory prediction, it achieved end-point errors below 8 m at 20 s in single-step, multi-step, and recursive forecasting, and accomplished reverse launch-point prediction for the first time with a total error of 8.31 m. Although focused on projectiles, its integration of short- and long-term dependencies, recursive refinement, and multi-step extrapolation closely parallels our 4D aircraft trajectory forecasting task and provides a direct reference for joint single- and multi-step trajectory modeling.

With the continuous advancement of deep learning networks, the issues arising from model complexity have gradually become inevitable challenges in this field. Among these, the enormous demand for computational resources is the most prominent. Knowledge distillation, as an efficient model compression method in the field of deep learning, has been widely applied in various scenarios. Gou et al. (2024) proposed a novel knowledge distillation approach, namely Forward and Feedback Knowledge Distillation (FFKD). Experimental results demonstrated that FFKD outperforms existing state-of-the-art knowledge distillation methods on five visual recognition datasets, showing great potential in deploying compact deep models in intelligent applications such as intelligent transportation, smart healthcare, and distributed intelligence. Xie et al. (2024) put forward a knowledge distillation method based on hierarchical feature logits. This method is particularly suitable for deployment on edge devices with limited memory and computing capabilities to achieve real-time decision-making and reduce data communication costs. Extensive experiments verified the effectiveness of the proposed model and proved its application potential in detecting internal defects of key components in electrical and mechanical systems within modern industry. Zhou et al. (2024) designed a model combining Graph Neural Networks (GNN) and Knowledge Distillation (KD), named Reconstructed Graph with Global–Local Distillation (RG-GLD), for lightweight anomaly detection in Internet of Things (IoT) communication networks. Through a graph network reconstruction strategy and a refined graph attention mechanism, the model effectively extracts and fuses features, thereby improving knowledge transfer efficiency. Experiments indicated that the RG-GLD model outperforms baseline methods in terms of knowledge transfer efficiency, classification accuracy, and computational load, making it suitable for deployment in sustainable IoT computing environments. Zhang et al. (2025) proposed a new Diversity-Enhanced Knowledge Distillation (DivKD) model for solving Mathematical Word Problems (MWP). This model adopts an adaptive diversity distillation method, enabling the student model to selectively transfer high-quality knowledge from the teacher model and learn to generate diverse mathematical equations. Experimental results on four MWP benchmark datasets showed that the DivKD model achieves higher answer accuracy than existing strong baseline models while maintaining high efficiency. Shen et al. (2025) addressed the issue of deploying large-scale GNN on resource-constrained devices and proposed a GNN knowledge distillation method based on adaptive meta-learning. This method allows the teacher model to dynamically update its parameters according to the learning feedback from the student model, thereby transferring knowledge more effectively. Additionally, a local structure preservation loss is introduced to avoid over-smoothing. Experimental results demonstrated that this method performs excellently in four benchmark tests, proving its effectiveness in reducing computational resource requirements while maintaining performance. Knowledge distillation and model compression technologies have become core means to achieve lightweight deep models. Through distillation, the representational capabilities of large-capacity teacher models are condensed into compact student networks, compressing the number of parameters and computational load several times without significant loss of accuracy. This directly reduces storage and power consumption, significantly alleviating the resource bottleneck of edge devices. Meanwhile, quantization and pruning can further eliminate redundancy at the bit level and structural level, collaborating with distillation to form a pipeline of “distillation followed by compression” or “alternating optimization.” This enables models to approach the theoretical limit of lightweight while maintaining task performance.

To address the issues of insufficient multi-factor feature extraction and excessive computational resource consumption in 4D trajectory prediction, this paper proposes an RCBAM-TCN-LSTM knowledge distillation model. This model consists of three main modules: the teacher model RCBAM, the student model TCN-LSTM, and the knowledge distillation mechanism between them. Each module works synergistically to achieve high-precision spatiotemporal coupling modeling of trajectory data.

1. Teacher model RCBAM: Based on the ResNet framework, it incorporates the CBAM mechanism with dual channel-spatial attention, enabling dynamic allocation of weights to different feature dimensions and key time windows in deep networks. Channel attention is responsible for highlighting important dimensions in multi-channel features, while spatial attention focuses on turning points and segments with drastic changes on the sequence timeline, thereby extracting more delicate spatial feature representations and providing high-quality soft label information for the student network.
2. Student model TCN-LSTM: It combines the advantages of TCN and LSTM. TCN captures multi-scale temporal patterns in parallel through multi-layer dilated convolutions and residual connections, while LSTM specializes in mining long-term dependencies within sequences.
3. Knowledge distillation mechanism: During the training process, the prediction distribution of the teacher model (soft labels) and the real labels (hard labels) are jointly used to construct the distillation loss, and the student network is guided through hybrid strategies such as cross-entropy or mean squared error. This mechanism not only smoothly transfers the deep spatial-channel-temporal coupling features of RCBAM to TCN-LSTM but also promotes the student network to optimize its overall expressive ability for complex trajectories while retaining its own advantages in temporal memory, achieving an optimal balance between lightweight performance and high precision.

The remaining parts of this paper are organized as follows. Section 2 describes the data sources and preprocessing. Section 3 elaborates on the basic knowledge of TCN, LSTM, ResNet, and CBAM networks. Section 4 presents the construction of the 4D trajectory model. Section 5 compares the performance of the main model through ablation experiments and conducts a comparative analysis with other mainstream baseline models. Finally, Section 6 summarizes the results and provides an outlook.

## 2 Data collection and preprocessing

### 2.1 ADS-B data

ADS-B historical trajectory data is the data basis for the 4D trajectory prediction in this paper. ADS-B is an aircraft operation monitoring technology, in which the transmitter of aircraft on-board equipment sends aircraft information to the ADS-B ground station or other aircraft loaded with ADS-B on-board equipment at a certain period, and the specific content includes: sampling time, position, altitude, speed, flight number, heading, climb or descent rate, etc. The data is collected from the ADS-B data, which is the data basis for the 4D trajectory prediction in this paper. The experimental data in this paper comes from Frequent Flyer Technology Company,^1^ and some data examples are shown in Table 1. As seen in Table 1, there are problems such as unequal sampling time intervals between neighboring trajectory points, and duplication of latitude and longitude data in different sampling points, which need to be preprocessed.

**Table 1:** Examples of ADS-B raw data.

Sampling time	Altitude/ft	Speed/kt	Direction/°	Latitude/°	Longitude/°
2022-11-07 t08:46:50	8,250	165	217	25.0771	102.9203
2022-11-07 t08:47:05	8,650	167	217	25.0706	102.9143
2022-11-07 t08:47:20	9,050	166	217	25.0641	102.9088
2022-11-07 t08:47:38	9,575	180	216	25.0559	102.9019
2022-11-07 t08:47:50	10,000	194	218	25.0679	102.8949
2022-11-07 t08:48:07	10,400	216	207	25.0378	102.8949
2022-11-07 t08:48:22	10,700	234	203	25.0263	102.8759

### 2.2 Data preprocessing

The preprocessing process includes steps such as outlier processing, track point interpolation, and normalization of the raw data, as well as data alignment and data construction.

#### 2.2.1 Outlier processing

Outliers are mainly categorized into three types: missing values, duplicates, and abnormal values. For missing points, the data is first sorted by flight number and timestamp. A record is deemed missing if the interval between adjacent records exceeds a preset threshold (set to 5 s in this study). When the proportion of missing points is low (<5%), linear interpolation is used to reconstruct fields such as longitude, latitude, and altitude. For longer missing segments, KNN interpolation (*k* = 3–5) can be combined for weighted smoothing. If excessive missing values lead to large interpolation errors, the entire trajectory is discarded.

Duplicate data is removed by comparing records with identical flight numbers, timestamps, and coordinates, or those with a distance <1 m and a time difference <0.5 s. Only the first observation is retained. For numerical anomalies, key indicators such as speed and climb rate are first evaluated using the Z-score (marked when |x–*μ*| > 3σ) to identify anomalies. Meanwhile, isolated jump points can be eliminated in the longitude-latitude-time space using simple distance-threshold-based clustering (the DBSCAN algorithm in this study, with eps = 0.001° and min_samples = 3). If the proportion of abnormal points is extremely low (<1%), they are replaced using Kalman filtering. However, if there are too many abnormal points or the duration of continuous anomalies exceeds 10 s, the trajectory is directly deleted to ensure the smoothness and reliability of subsequent model inputs.

#### 2.2.2 Track point interpolation and data alignment

**Figure 1:**
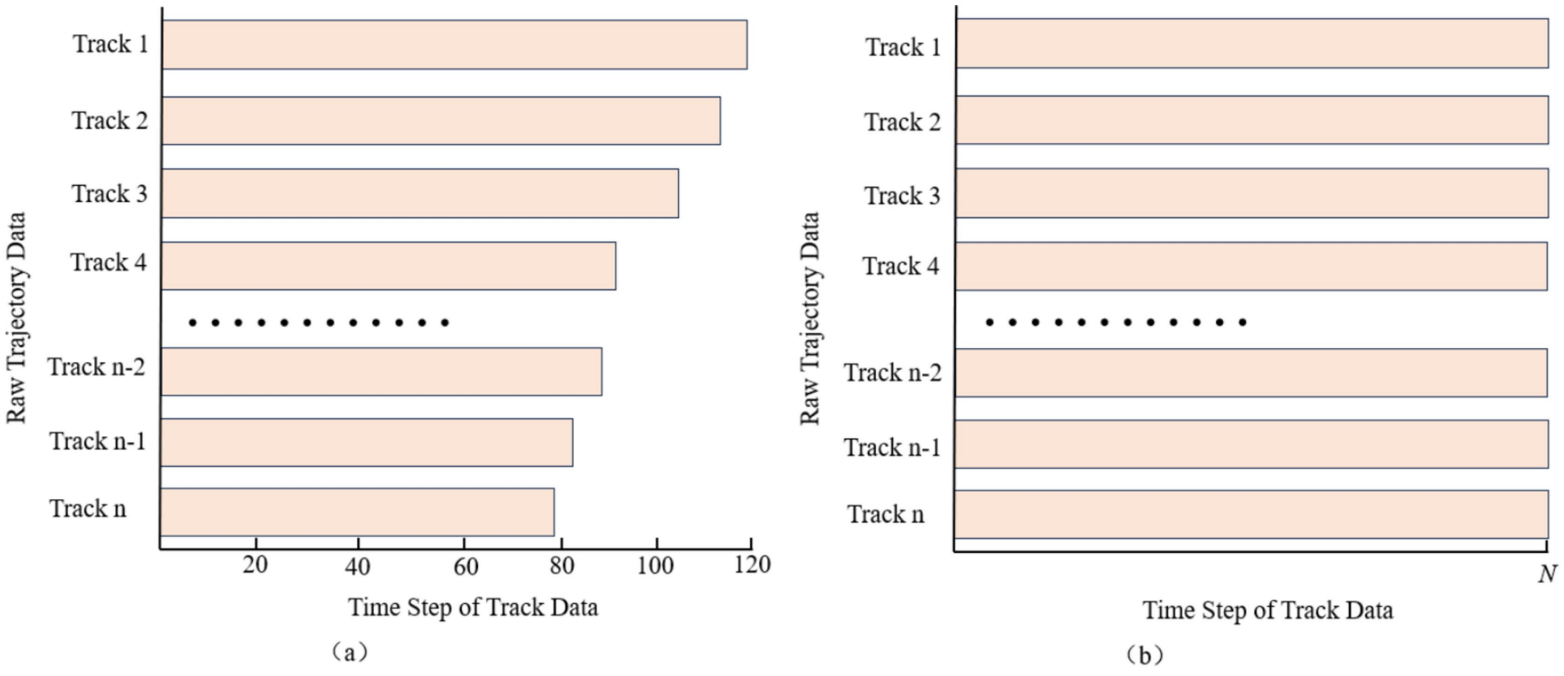
Comparison of data alignment pre-processing for trajectory data. **(a)** Unaligned data. **(b)** Undergone data alignment.

To address the problem of non-uniform temporal intervals in historical trajectory records, we reconstruct each trajectory via cubic-spline interpolation, enforcing an identical sampling frequency so that every pair of consecutive points is separated by the same time span. After interpolation, all trajectories share this uniform interval; however, because the total flight times differ, the resulting trajectories contain different numbers of points—that is, they have unequal sequence lengths, as shown in Figure 1a. To capture the overall trend of each trajectory, we then uniformly resample N points along the entire interpolated path, thereby aligning all sequences to the same fixed length and satisfying the input requirement of a uniform step count for the trajectory-prediction model, as illustrated in Figure 1b.

#### 2.2.3 Normalization

The trajectory data consists of different feature sequences, and the features have different scales and large differences in the data range, which may cause the model to be more sensitive to some features and less sensitive to other features, making it difficult for the training process to converge. Therefore, in this section, in order to avoid this situation from affecting the prediction results, the trajectory data need to be normalized. By using normalization, the training process can be stabilized, the convergence speed of the model can be improved, the occurrence probability of gradient explosion and gradient disappearance can be reduced, and the generalization ability and prediction accuracy of the model can be improved. This model uses min-max normalization in training to map all values between (0,1), and the normalization formula is as follows in Equation 1:


(1) $$\text{Normalized}(x)=\frac{x-x_{\min}}{x_{\max}-x_{\min}}$$

Where Normalized is the normalization result, X is the input value of the independent variable, and max and min are the maximum and minimum values of the corresponding input tensor, respectively.

#### 2.2.4 Data construction

In this section, we first regard the historical flight trajectory dataset $\left\{D_{1},D_{2},\ldots ,D_{m}\right\}$ as a whole composed of a sequence of Features-dimensional feature vectors. Fixed-length subsequences with (*Time-step*) are sequentially extracted as model inputs using a sliding window method. For single-step prediction scenarios, the trajectory point at the moment immediately following the end of the window serves as the label. After receiving the input tensor with the shape (*Batch-size, Time-step, Features*), the network only needs to compress and aggregate the spatiotemporal features captured within the time window through a mapping layer—typically a fully connected layer or a sequence-to-scalar output module—and output the next-moment position prediction with the shape (*Batch-size, 1, Features*) or (*Batch-size, Features*). This design enables the model to focus on extracting the most valuable feature mappings for a single future point from the information of the past *Time-step*.

In multi-step prediction scenarios, the sliding window of the same length is required to predict not only the trajectory point at the next moment after each movement but also output the complete trajectory sequence for the subsequent *H* time steps simultaneously. To this end, the model structure usually incorporates a sequence-to-sequence multi-output branch design at the end of the network to ensure that the time dimension of the output tensor is consistent with the *H*-step length expected by the label. Specifically, the encoder part receives the input tensor and extracts high-dimensional representations through a series of temporal convolutions or recurrent units. In the decoder or multi-branch output layer, the model generates or parallelly outputs the trajectory features for the future *H* steps based on these representations. The final shape of the output tensor is (*Batch-size, H, Features*), which is consistent with the real label. During the training process, single-step prediction typically only requires calculating the mean squared error or cross-entropy loss once, while multi-step prediction needs to weight or overall calculate the errors of the *H* time steps to balance the prediction accuracy of both short-term and slightly long-term periods.

**Figure 2:**
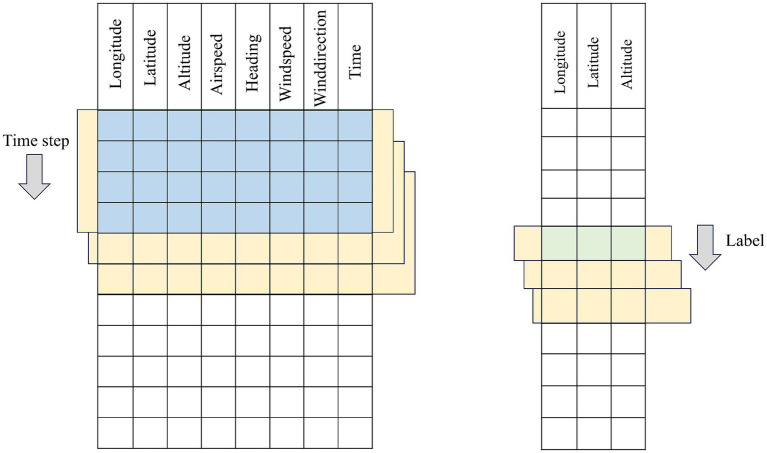
Schematic diagram of single step time series construction.

**Figure 3:**
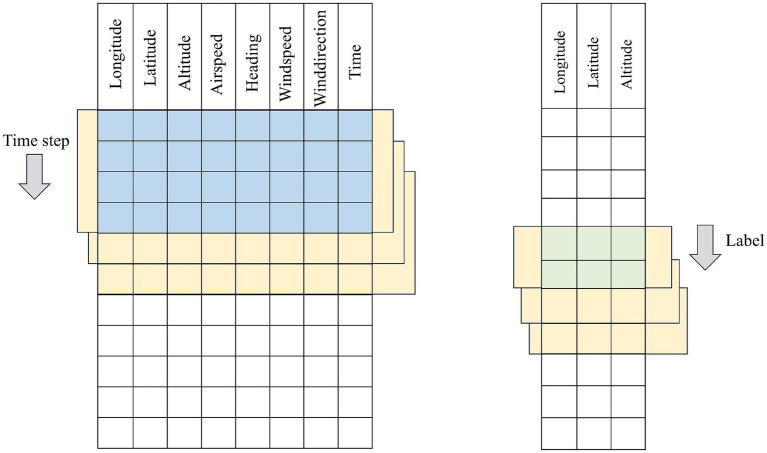
Multi-step time series construction principle diagram.

In the data construction phase, regardless of single-step or multi-step prediction, the window starts from the first row and slides backward with a step size of 1 until covering the entire trajectory sequence, thereby generating a sufficient number of training samples. The core differences between single-step and multi-step prediction lie in the label selection method, model output dimension, and design of the network tail: the former focuses on single-point mapping with small and simple output dimensions; the latter needs to have the ability to generate or parallelly output multi-step sequences and simultaneously consider the overall error of these *H* steps in the training loss function. Figures 2, 3 respectively show the intuitive illustrations of these two different label construction and network output frameworks. In this way, we not only maintain the consistency of the data construction process but also clearly distinguish the procedures and architectural differences between single-step and multi-step predictions at the model structure level.

## 3 Basic algorithm principle

### 3.1 Long short-term memory network (LSTM)

LSTM is an improved model of RNN for solving the problem of gradient vanishing and gradient explosion. The LSTM model contains three main gates, which are forgetting gate, memory gate and output gate.

The operational steps of LSTM are as follows:

The forgetting gate is used to determine whether the information in the cell state of the previous time step needs to be forgottenas shown in Equation 2:


(2) $$f_{t}=\sigma (W_{f}\times [y_{t-1}\cdot x_{t}]+b_{f})$$

The input gate is used to determine the new information added to the cell state. $\tilde{S_{t}}$ is the candidate value generated by the input gate $i_{t}$ , as shown in Equation 3 and Equation 4:


(3) $$i_{t}=\sigma (W_{i}\times [y_{t-1},x_{t}]+b_{i})$$

(4) $$\tilde{S_{t}}=\tanh(W_{c}\times [y_{t-1},x_{t}]+b_{c})$$

Combining the outputs of the forgetting gate and the input gate updates the cell state as shown in Equation 5:


(5) $$S_{t}=f_{t}⊙S_{t-1}+i_{t}⊙\tilde{S_{t}}$$

The output gate, outputs the cell state value; $y_{t}$ is the hidden state output, as shown in Equation 6 and Equation 7:


(6) $$o_{t}=\sigma (W_{o}[y_{t-1},x_{t}]+b_{o})$$

(7) $$y_{t}=o_{t}⊙\tanh(S_{t})$$

**Figure 4:**
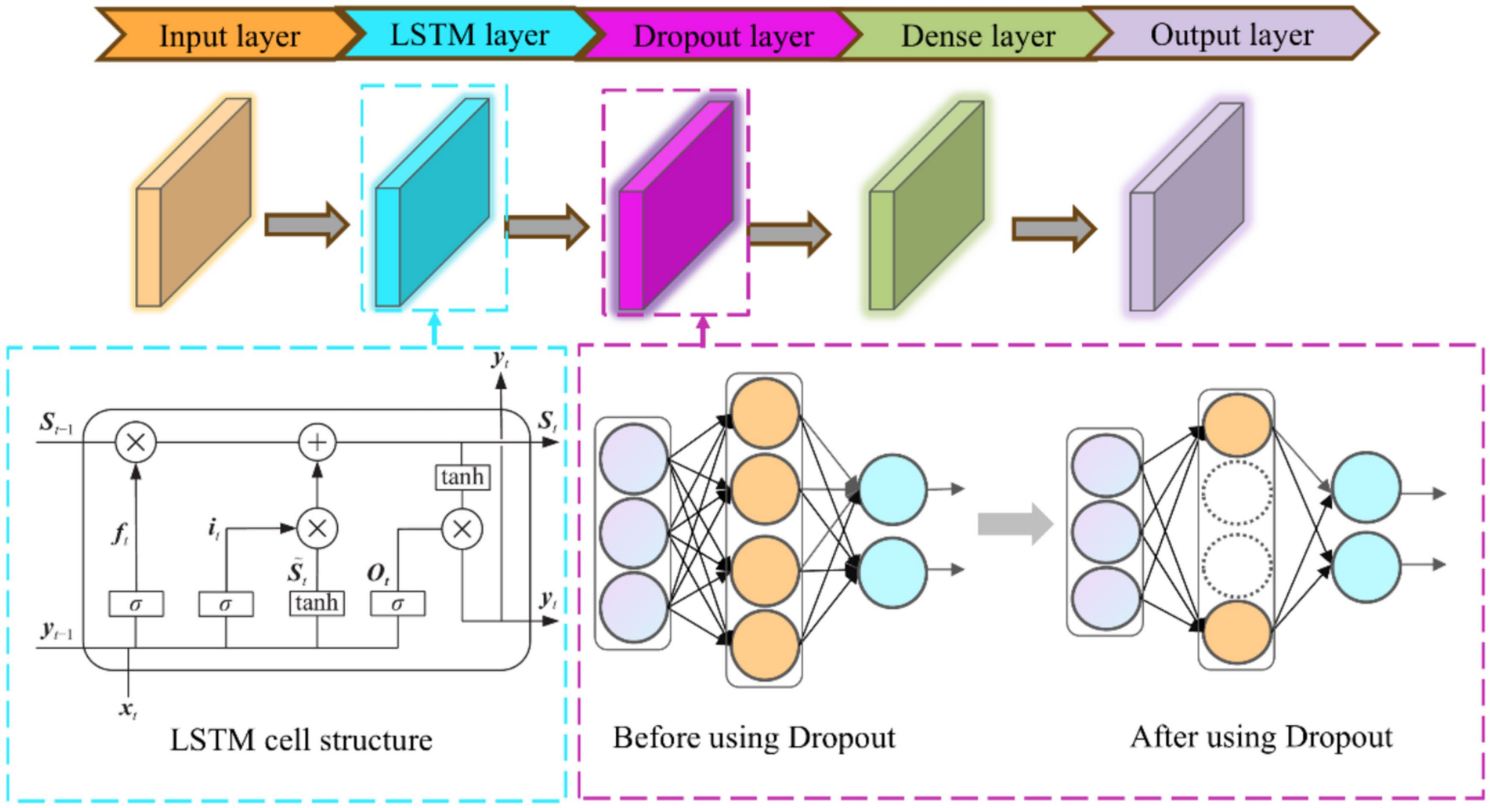
LSTM network structure diagram.

Where $i_{t}$ , $f_{t}$ , $o_{t}$ are the input gate, forgetting gate and output gate respectively; where $y_{t-1}$ denotes the previous cell output and $x_{t}$ denotes the current cell input; *σ* denotes the sigmoid activation function; W and b are the weight coefficients and the bias function, respectively; $\tilde{S_{t}}$ and $S_{t}$ are the candidate memory cell state and the memory cell state, respectively; *tanh* is the hyperbolic tangent activation function. The architecture of the proposed LSTM model is shown in Figure 4.

### 3.2 Time convolutional network (TCN)

**Figure 5:**
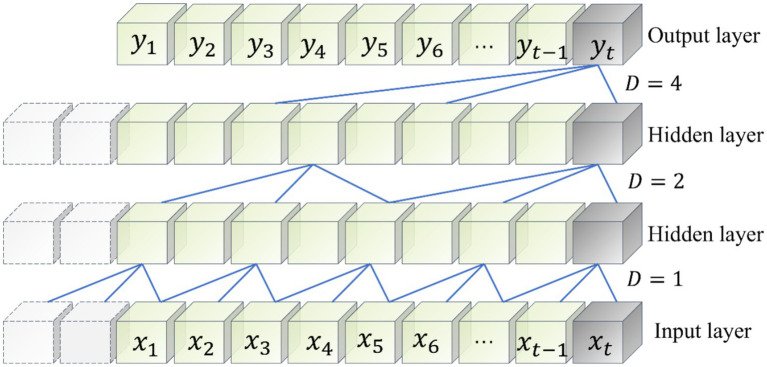
TCN expansion of causal convolution structure.

Temporal Convolutional Network is an improved novel network based on CNN proposed by Bai et al. (2018), which enables TCN network to capture features in time series data more efficiently due to its special dilation causal convolution and residual module, which makes TCN network have longer time dependence compared to CNN. The structure of dilation causal convolution with dilation coefficients D of 1, 2 and 4 in TCN is shown in Figure 5.

In depthwise convolution, a separate convolutional filter is applied to each input channel. If the input consists of M state features, there are M input channels and thus M filters, each operating on its corresponding channel independently. This produces one feature map per input feature. Pointwise Convolution then follows, using a 1 × 1 kernel to merge information across these channels. While it does not expand spatial dimensions, it effectively integrates the per-channel features into a richer, combined representation. By decomposing a standard convolution into these two steps, depthwise separable convolutions dramatically reduce both parameter count and computational cost—eliminating the need for full connections between all input and output channels at every spatial location. Moreover, the reduced parameterization helps mitigate overfitting and makes the model well suited for deployment on resource-constrained devices.

### 3.3 ResNet network

Residual Network (ResNet) is a deep convolutional neural network architecture that solves the problem of training difficulties as the depth of the network increases, in particular, the problems of gradient vanishing and gradient explosion. The core idea of ResNet is the introduction of Residual Learning. Traditional neural network layers are directly fitted with a mapping relationship between the bottom input x and the top output F(x), i.e., y = F(x). Such a mapping can lead to the introduction of errors, especially if the number of network layers is large and the network is deep. In contrast, ResNet lets each layer of the network learn the residual mapping, i.e., the difference between the input and the output. If the input is x, the residual learning part is F(x), and y is the output, when F(x) learns a residual close to zero, then y is close to x. The basic blocks of ResNet can be represented in Equation 8 as follows:


(8) y=F(x)+x

The basic building blocks of ResNet are residual blocks. Each residual block contains two or three convolutional layers, and a skip connection that skips over these layers. This connection is made by simply adding the inputs of the block to its outputs, allowing the gradient of the deep network to also pass directly through these skip connections. During training, the gradient is propagated back through both F(x) and x paths by the backpropagation algorithm. If F(x) learns a residual close to zero, then this residual will have a small effect on the gradient propagation, thus avoiding the gradient vanishing problem. In summary, by introducing the residual learning mechanism, ResNet enables the network to maintain convergence even if more levels are added, effectively solving the gradient vanishing and gradient explosion problems in deep neural network training, thus realizing the construction of deeper network structures and the extraction of deeper features.

### 3.4 CBAM network

**Figure 6:**
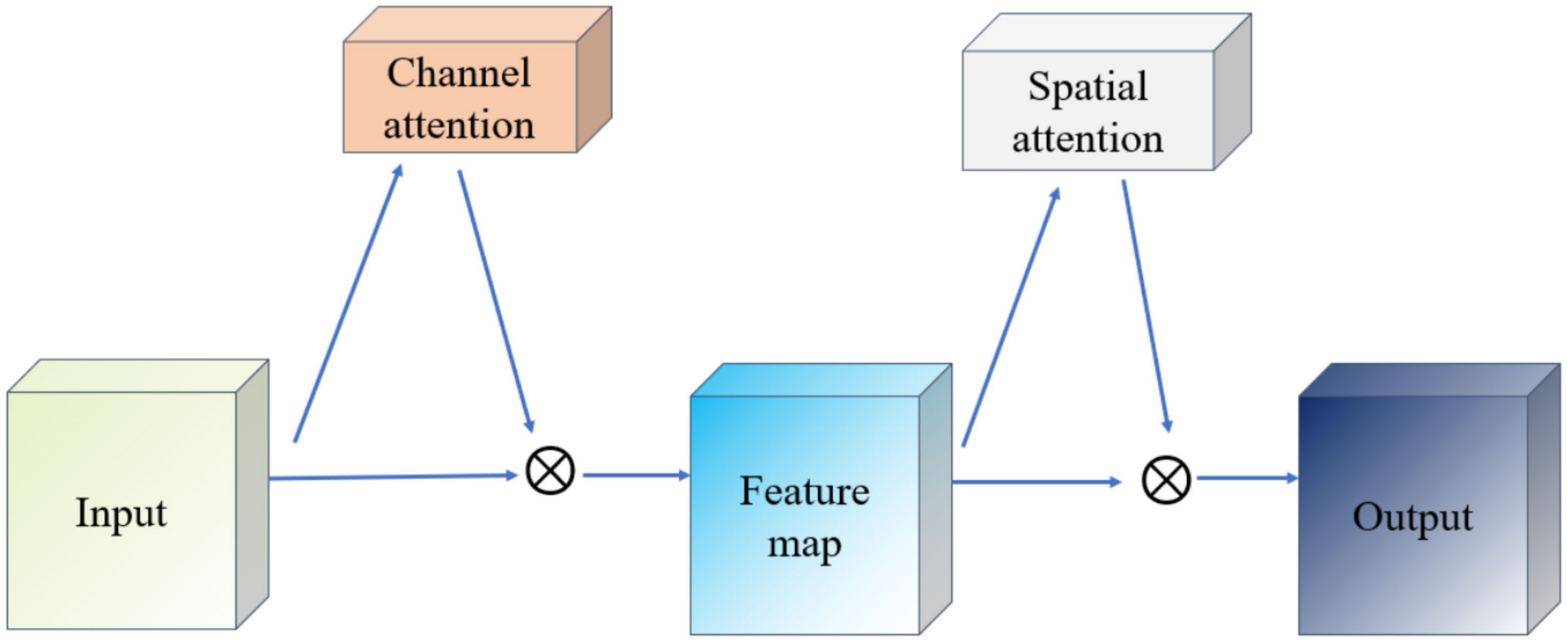
Schematic diagram of CBAM network.

The Convolutional Block Attention Module (CBAM) is a lightweight mechanism that enhances feature representations in convolutional neural networks by sequentially applying channel and spatial attention. As illustrated in Figure 6, CBAM first routes the input feature map through the Channel Attention module, which computes per-channel weights based on pooled descriptors and reweights each channel accordingly. The reweighted feature map is then fed into the Spatial Attention module, which generates a spatial mask by aggregating channel-wise information and highlights important spatial regions. Finally, both attention maps are element-wise multiplied with the original feature map to yield an adaptively refined representation. This two-stage attention process enables the network to focus on both “what” (channel importance) and “where” (spatial importance), thereby improving its ability to capture salient information.

**Figure 7:**
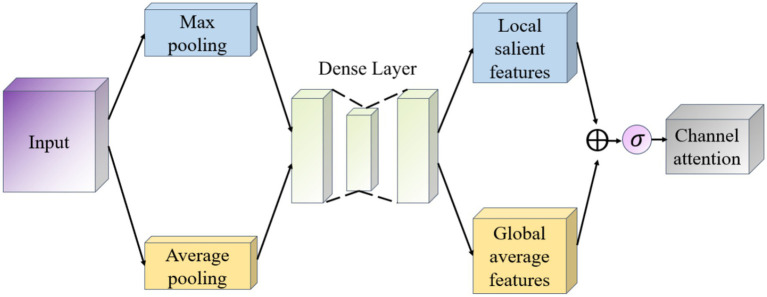
Schematic diagram of the channel attention module.

The goal of the Channel Attention module (Channel Attention) is to determine the importance of individual channels (i.e., different feature maps), which works as shown in Figure 7. It first uses global average pooling and global maximum pooling operations to generate two different feature maps, which capture the distribution information of the channels, respectively. Then, these two feature maps are fed into a shared fully connected layer, which contains a hidden layer. Finally, the outputs of these two MLPs are summed by elements and a Sigmoid activation function is applied to obtain the attention weights for each channel.

**Figure 8:**
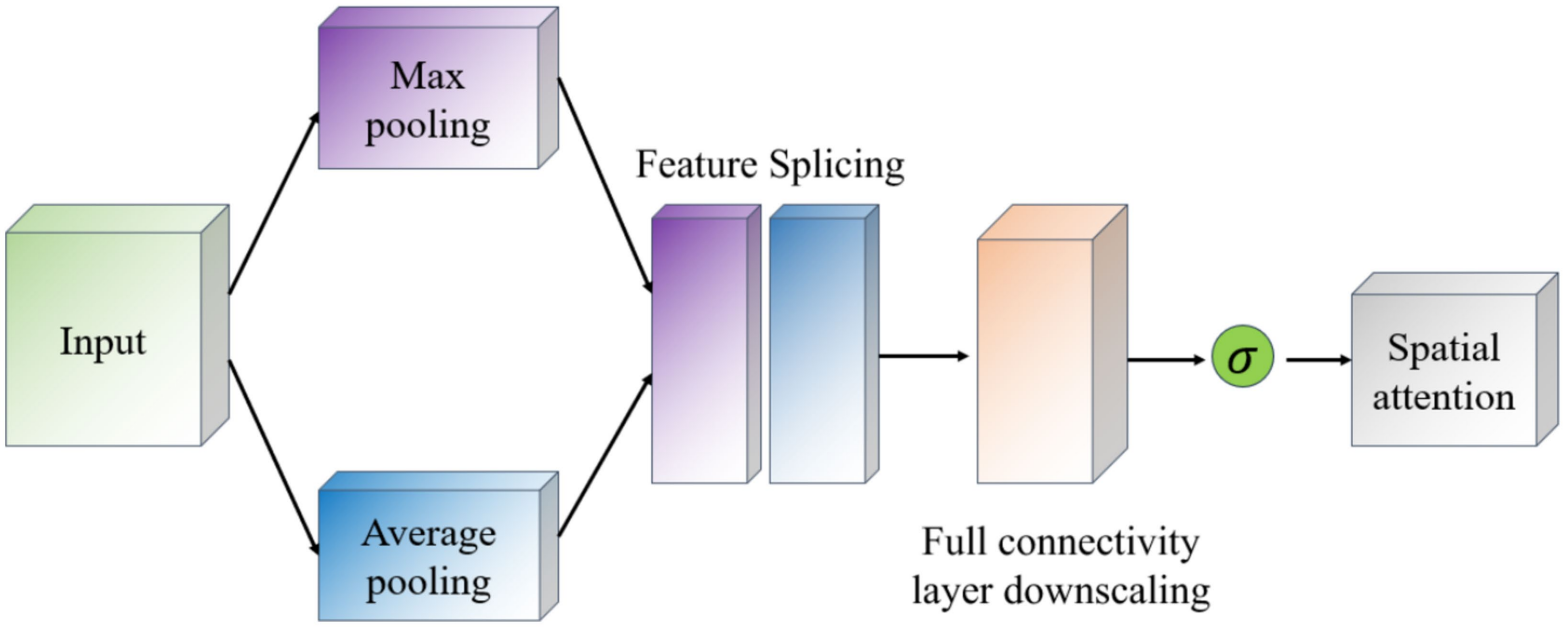
Schematic diagram of the spatial attention module.

The Spatial Attention module (Spatial Attention) follows the Channel Attention and its purpose is to highlight important regions in the spatial dimension and works as shown in Figure 8. This module uses the output of channel attention and processes it to generate a two-dimensional attention map. Specifically, it first performs average and maximum pooling in the channel direction on the input feature maps to generate two 2D feature maps, which are then stitched together in the channel dimension and passed through a convolutional layer to produce the final spatial attention map. This attention feature map is also activated by a Sigmoid function in order to weight the original input feature map.

## 4 Teacher model—student model predictive model

### 4.1 Student model (TCN-LSTM)

**Figure 9:**
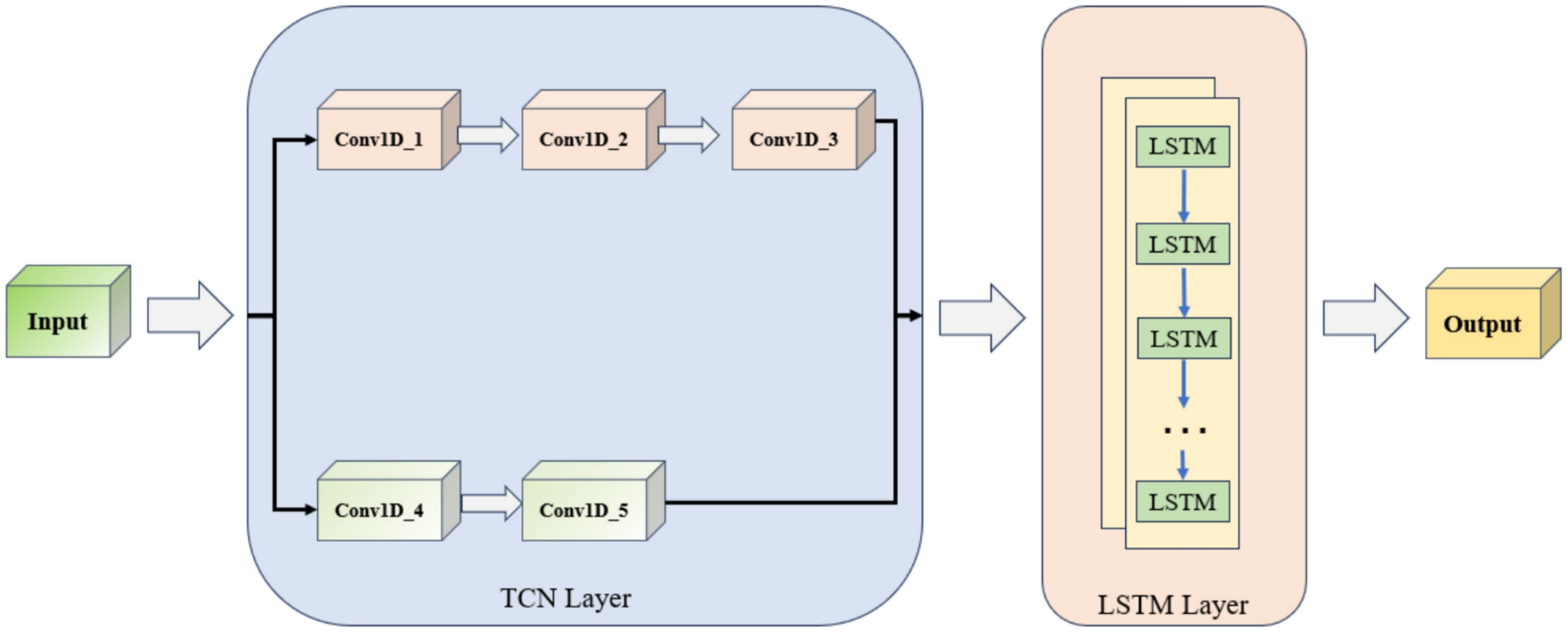
TCN-LSTM network structure.

The TCN-LSTM model prediction process established in this paper is shown in Figure 9. The data enter the TCN layer from the input layer, which consists of five one-dimensional convolutional neural networks (Conv1D), of which Conv1D_4 and Conv1D_5 belong to the residual block. The TCN layer is connected to two LSTM layers after the TCN layer, and finally accesses to the output layer. The TCN layer can efficiently capture the local features of the time-series data, and the LSTM layer is responsible for modeling the long-term dependency, which together constitute the TCN-LSTM model. Constitute the main framework of the TCN-LSTM model.

### 4.2 Teacher model (ResNet-CBAM)

**Figure 10:**
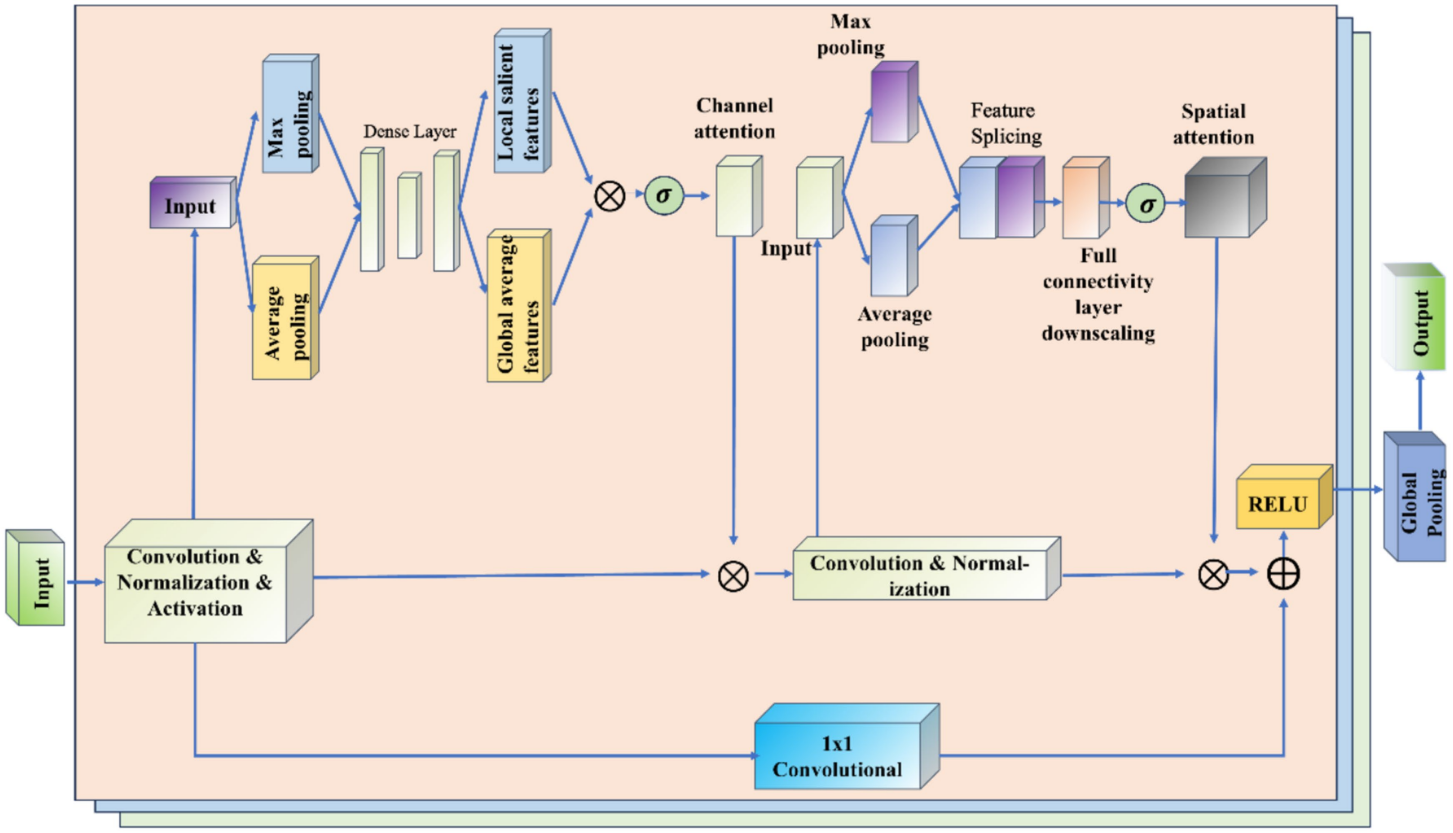
Working schematic of the RCBAM model.

The RCBAM model is essentially a combination of a convolutional neural network and an attention mechanism, aiming at feature extraction and prediction of the trajectory data from a spatial and state change perspective. Figure 10 shows the working schematic of the RCBAM model.

The role of ResNet is to efficiently extract high-level features from flight trajectory data using a deep structure. By treating the sequence data as a one-dimensional image (time as width), the change in features over time is captured. Meanwhile residual learning is introduced to solve the problem of overfitting and network degradation that deep learning models are prone to when dealing with complex time series prediction tasks, and to solve the problem of gradient vanishing and gradient explosion in deep networks. The role of CBAM’s channel attention is to allow the model to dynamically assign weights to each input feature, thereby highlighting the features that will be most helpful for prediction. The role of CBAM’s spatial attention is to dynamically assign weights to each specific point in time, thereby highlighting the time windows that are most helpful for prediction. The combination of the two CBAM attention mechanisms allows the model to identify both important feature channels and also features at important moments in the trajectory sequence. This helps to build more refined prediction models that take into account both time dependence and feature importance.

### 4.3 Model working principle

**Figure 11:**
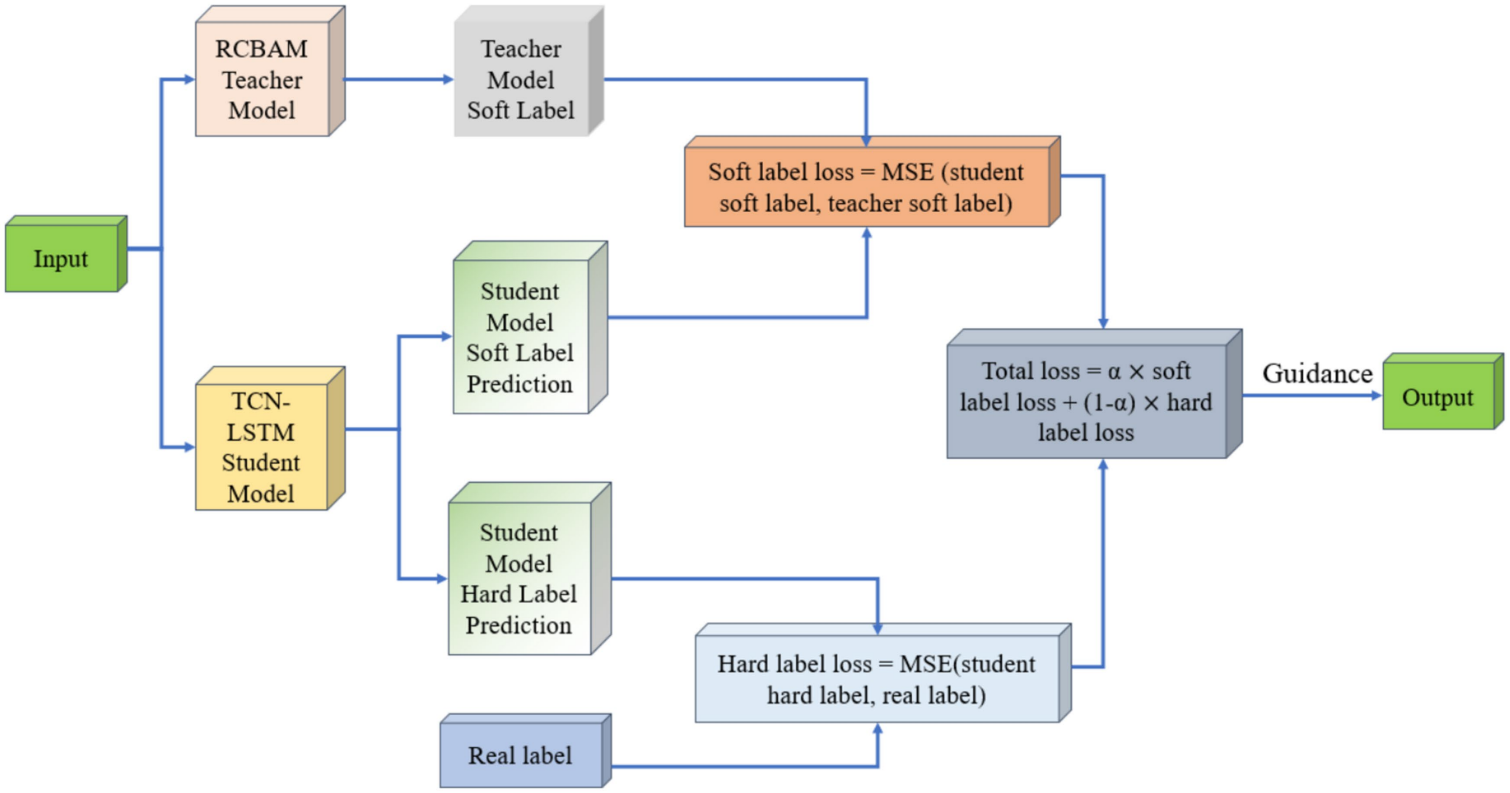
Schematic diagram of knowledge distillation model based on RCBAM-TCN-LSTM.

The student network is implemented as a lightweight TCN-LSTM, which has a total of 823,423 parameters (573,213 trainable and 348,210 non-trainable), roughly one-fifth the size of the RCBAM teacher. Its simple combination of dilated causal convolutions and a two-layer LSTM makes it an ideal candidate for efficient temporal modeling. Figure 11 illustrates the overall architecture of the RCBAM-TCN-LSTM framework.

In our distillation scheme, the high-capacity RCBAM model serves as the teacher, supplying “soft labels” in the form of its predicted longitude, latitude, altitude, speed, and heading. These soft labels act as smooth, auxiliary supervisory signals that convey the teacher’s learned data distributions and help regularize the student, mitigating overfitting. Concurrently, the true trajectory measurements provide “hard labels,” offering precise, observation-based targets that the student is explicitly trained to match. During training, we compute two mean-squared-error losses: one between the student’s outputs and the soft labels, and another between the student’s outputs and the hard labels. A weighted sum of these two losses forms the total training objective, balancing guidance from both the teacher’s predictions and the real data. This joint-supervision strategy leverages the complementary strengths of model-based and data-driven signals to significantly enhance the student’s predictive performance.

## 5 Simulation verification and analysis

### 5.1 Experimental data and experimental environment

The trajectory dataset used in this paper is the real ADS-B historical trajectory data of inbound flights at Zhuhai Jinwan Airport, which retains the trajectory features such as time, speed, altitude, longitude, latitude, etc., and the dataset is stored in the form of csv. Based on this dataset, a 4D trajectory prediction model based on neural network is constructed for trajectory feature and position prediction. From the whole dataset, about 2,842 complete trajectories, totaling 288,200 trajectory points, were intercepted, screened, and retained. All the data were divided into training set, validation set and test set according to the ratio of 8:1:1.

The experimental equipment is a laboratory desktop computer with Intel(R) Core(TM) i7-10700 CPU @ 2.90 GHz, 2.90 GHz, and 16 GB of RAM on board.

### 5.2 Model evaluation metrics

In order to compare the performance of different algorithms, this paper uses the Root Mean Square Error (RMSE), Mean Absolute Error (MAE) and Mean Absolute Percentage Error (MAPE) and the Coefficient of Determination (*R*^2^) were used as evaluation indexes. Among them, the smaller the values of MAE, RMSE and MAPE are, the better the model prediction effect is; the closer the value of *R*^2^ is to 1, the better the fitting effect of the prediction model is. The specific calculation formula is as follows in Equations 9-12:


(9) $$\text{RMSE}=\sqrt{\frac{1}{n}\sum_{i=1}^{n}{\left(y_{i}-Y_{i}\right)}^{2}}$$

(10) $$\mathrm{MAE}=\frac{1}{n}\sum_{i=1}^{n}|y_{i}-Y_{i}|$$

(11) $$\text{MAPE}=\frac{1}{n}\sum_{i=1}^{n}|\frac{y_{i}-Y_{i}}{y_{i}}|\times 100\%$$

(12) $$R^{2}=1-\frac{\sum_{i=1}^{n}{\left(y_{i}-Y_{i}\right)}^{2}}{\sum_{i=1}^{n}{\left(y_{i}-\bar{y_{i}}\right)}^{2}}$$

Where n is the number of samples, $y_{i}$ is the actual value of the samples, $Y_{i}$ is the predicted value of the model, and $\bar{y_{i}}$ is the summed average of the actual values of the samples.

### 5.3 Model parameter settings

#### 5.3.1 Student model

The TCN-LSTM hybrid network consists of five stacked 1D dilated convolutional layers followed by a two-layer LSTM module (see Table 2 for hyperparameters).

**Table 2:** Main hyperparameters of TCN-LSTM model.

Network type	Number of channels	Convolutional kernel size	Expansion factor	Activation function
Conv1D_1	16	3	1	Relu
Conv1D_2	8	3	1	Relu
Conv1D_3	4	3	1	Relu
Conv1D_4 (residual block)	8	3	1	Relu
Conv1D_5 (residual block)	4	3	1	Relu
LSTM_1	8	—	—	Relu
LSTM_2	8	—	—	Relu

In the convolutional block, Conv1D_1 through Conv1D_3 successively reduce the channel count from 16 to 8 to 4, each using a kernel size of 3, dilation factor of 1, and ReLU activation to ensure strong nonlinear representation and rapid convergence. Conv1D_4 and Conv1D_5 then maintain 8 and 4 channels respectively, with identical kernel and dilation settings, and introduce residual connections at each layer. These skip links preserve low-level features and mitigate vanishing gradients as the network deepens. After extracting and fusing multi-scale temporal features, the output is fed into the LSTM block. Both LSTM_1 and LSTM_2 are configured with 8 hidden units and ReLU activations, balancing efficient gating with the capacity to model long-term dependencies. Stacking two LSTM layers further deepens the model’s representational power.

Overall, the convolutional layers focus on parallelized local pattern extraction—with residual paths to reinforce information flow—while the LSTM layers handle dynamic sequence memory. The uniform kernel size and activation choice simplify tuning, and the progressive channel reduction controls model size without sacrificing expressiveness. This design strikes a practical balance between accuracy and computational efficiency for medium-length sequence forecasting.

#### 5.3.2 Teacher model

The parameter configuration of the hyperparameters is critical for model training and directly affects the learning ability and prediction performance of the model. The parameters of the model must be carefully set and adjusted prior to trajectory prediction to ensure that the model can effectively learn from the data and make accurate predictions. These parameters include, but are not limited to, the window length, the number of filters, the learning rate, and the number of iterations. Table 3 details the important parameters that need to be set during the model training process, and the selection of each parameter is based on extensive experiments and analyses, aiming to provide an optimal model training environment for the trajectory prediction task.

**Table 3:** Parameter configuration of RCBAM model.

Parameter name	Parameter value
Optimizer	Adam
Batch size	32
Learning rate	0.0005
Optimizer	Adam
Number of epochs	50
Window length	32
Input shape	(32,6)
Output shape	3
Residual block configuration	[2,2,2]
Number of filters	[64,128,256,512]

#### 5.3.3 Pseudocode

Algorithm 1 shows the RCBAM-TCN-LSTM Knowledge-Distillation 4D Trajectory Prediction.

**ALGORITHM 1:**
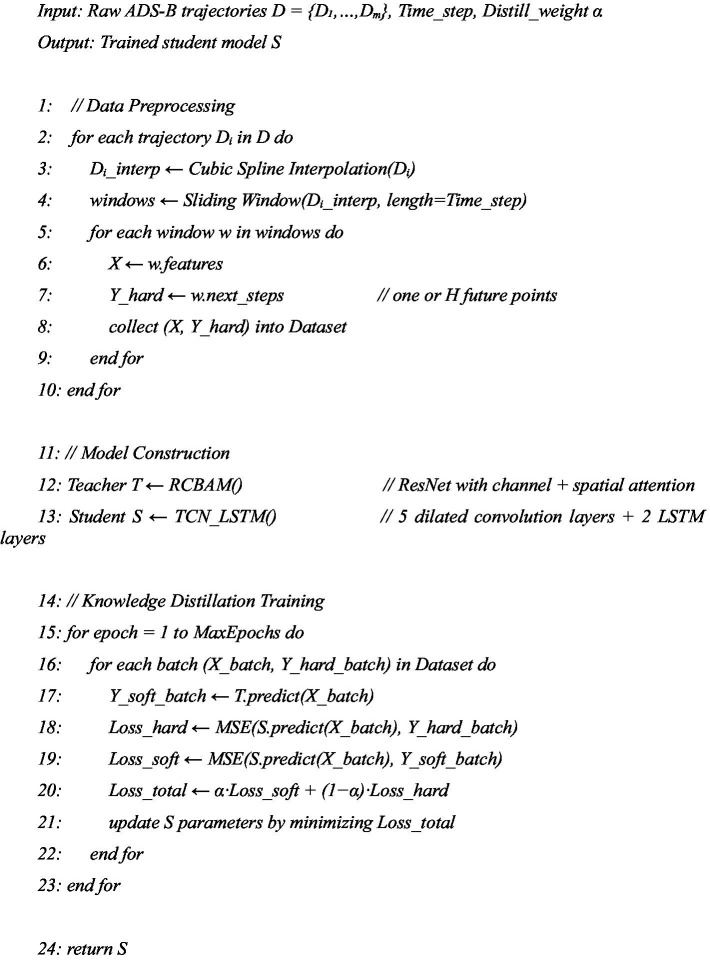
RCBAM-TCN-LSTM knowledge-distillation 4D trajectory prediction.

### 5.4 Comparison of ablation experiments

In the single-step prediction scenario (Table 4), the distilled RCBAM-TCN-LSTM model markedly outperforms both the original RCBAM teacher and the TCN-LSTM student. Specifically, its MAE in longitude, latitude, and altitude decreases to 0.0415°, 0.0456°, and 264.9 ft—representing reductions of approximately 32, 36, and 48% relative to the teacher (0.0613°, 0.0711°, 509.3 ft) and 55–58% relative to the student (0.0913°, 0.1032°, 632.5 ft). RMSE follows a similar trend, with the distilled model achieving 0.0411°/0.0466°/265.8 ft. (42–46% lower than the teacher, >55% lower than the student). In terms of MAPE, errors fall to 6.44, 8.66, and 21.37%, compared with 11.94–63.44% for the teacher and up to 85.32% for the student, while *R*^2^ improves from 0.9343–0.9355–0.9211 to 0.9867–0.9794–0.9675, confirming the distilled model’s superior integration of spatial attention and temporal dynamics.

**Table 4:** Comparison of RCBAM-TCN-LSTM knowledge distillation model indicators.

Time step	Evaluation metrics	Prediction model	Longitude/°	Latitude/°	Altitude/ft
Single-step	MAE	TCN	0.1853	0.1743	831.5
		TCN-LSTM	0.0913	0.1032	632.5
		RCBAM	0.0613	0.0711	509.3
		RCBAM-LSTM	0.6002	0.0684	431.5
		Teacher-Student Model	0.0415	0.0456	264.9
	RMSE	TCN	0.2193	0.1843	753.5
		TCN-LSTM	0.0926	0.1151	599.1
		RCBAM	0.0716	0.0828	495.6
		RCBAM-LSTM	0.6732	0.0742	475.5
		Teacher-Student Model	0.0411	0.0466	265.8
	MAPE	TCN	0.2142	0.8532	0.8984
		TCN-LSTM	0.1231	0.6311	0.8532
		RCBAM	0.1194	0.3432	0.6344
		RCBAM-LSTM	0.1134	0.4891	0.8219
		Teacher-Student Model	0.0644	0.0866	0.2137
	*R* ^2^	TCN	0.7432	0.7443	0.7321
		TCN-LSTM	0.8322	0.8132	0.7753
		RCBAM	0.9343	0.9355	0.9211
		RCBAM-LSTM	0.9456	0.9378	0.9324
		Teacher-Student Model	0.9867	0.9794	0.9675
Multi-step	MAE	TCN	0.1054	0.1424	621.6
		TCN-LSTM	0.0797	0.0684	544.8
		RCBAM	0.0688	0.0645	534.5
		RCBAM-LSTM	0.0563	0.0521	397.3
		Teacher-Student Model	0.0378	0.0256	237.6
	RMSE	TCN	0.1532	0.1288	682.1
		TCN-LSTM	0.0867	0.0955	565.4
		RCBAM	0.0663	0.0746	455.4
		RCBAM-LSTM	0.0432	0.0489	405.3
		Teacher-Student Model	0.0211	0.0367	203.7
	MAPE	TCN	0.1343	0.7422	0.8422
		TCN-LSTM	0.1077	0.2789	0.5323
		RCBAM	0.0977	0.1889	0.5323
		RCBAM-LSTM	0.0843	0.1942	0.4211
		Teacher-Student Model	0.0475	0.0432	0.1343
	*R* ^2^	TCN	0.7742	0.8022	0.8223
		TCN-LSTM	0.8477	0.8521	0.8212
		RCBAM	0.9475	0.9374	0.9482
		RCBAM-LSTM	0.9423	0.9354	0.9321
		Teacher-Student Model	0.9932	0.9798	0.9833

Under multi-step prediction, the advantages of the distilled model are further amplified. Its MAE decreases to 0.0378°, 0.0256°, and 237.6 ft—corresponding to additional reductions of 9, 44, and 10% relative to its single-step performance—whereas the teacher and student models remain above 0.0645°/534.5 ft. and 0.0684°/544.8 ft., respectively. RMSE declines from 0.0411°/0.0466°/265.8 ft. to 0.0211°/0.0367°/203.7 ft. (49%/21%/23% further improvement), and MAPE drops to 4.75, 4.32, and 13.43% (reductions of 26–50% from single-step). *R*^2^ also increases to 0.9932, 0.9798, and 0.9833, whereas both teacher and student models fail to exceed *R*^2^ = 0.95 in this setting. These results demonstrate that, rather than suffering from error accumulation, our distilled model leverages extended context and global sequence information to achieve higher accuracy and robustness in multi-step forecasting.

In evaluating our trajectory prediction models, we not only compared the overall performance of the full model against the RCBAM teacher and the TCN-LSTM student, but also conducted systematic ablation studies to isolate each component’s contribution. We introduced an intermediate RCBAM-LSTM variant to quantify the individual effects of spatial attention and temporal modeling. The ablation results for single-step forecasting show that the RCBAM-LSTM model (channel-and-spatial attention followed by LSTM) reduces MAE by approximately 40% compared to the baseline TCN. Adding the TCN structure on top of RCBAM-LSTM (i.e., the TCN-LSTM variant) further reduces error by another 12% through multi-scale temporal feature extraction. The distilled RCBAM-TCN-LSTM model then builds on these gains: knowledge distillation lowers MAE by an additional 40–60% on average and raises *R*^2^ above 0.98. Concretely, latitude MAE drops from 0.185° with the TCN to 0.091° with TCN-LSTM, then to 0.060° with RCBAM-LSTM, and finally to 0.041° with the distilled model. In multi-step forecasting, the ablations similarly confirm cumulative benefits: RCBAM-LSTM improves resistance to error accumulation by about 20%, TCN-LSTM adds a further 15% reduction in long-term dependency error, and the distilled model—leveraging both—lowers overall MAE by roughly 40% compared to RCBAM-LSTM and boosts *R*^2^ by 0.02–0.05.

By including the RCBAM-LSTM control group, we clearly quantify how the spatial attention module and the temporal convolution module each enhance predictive accuracy, and demonstrate how knowledge distillation synergistically fuses their strengths. These findings provide strong empirical guidance for model design and architectural optimization.

**Figure 12:**
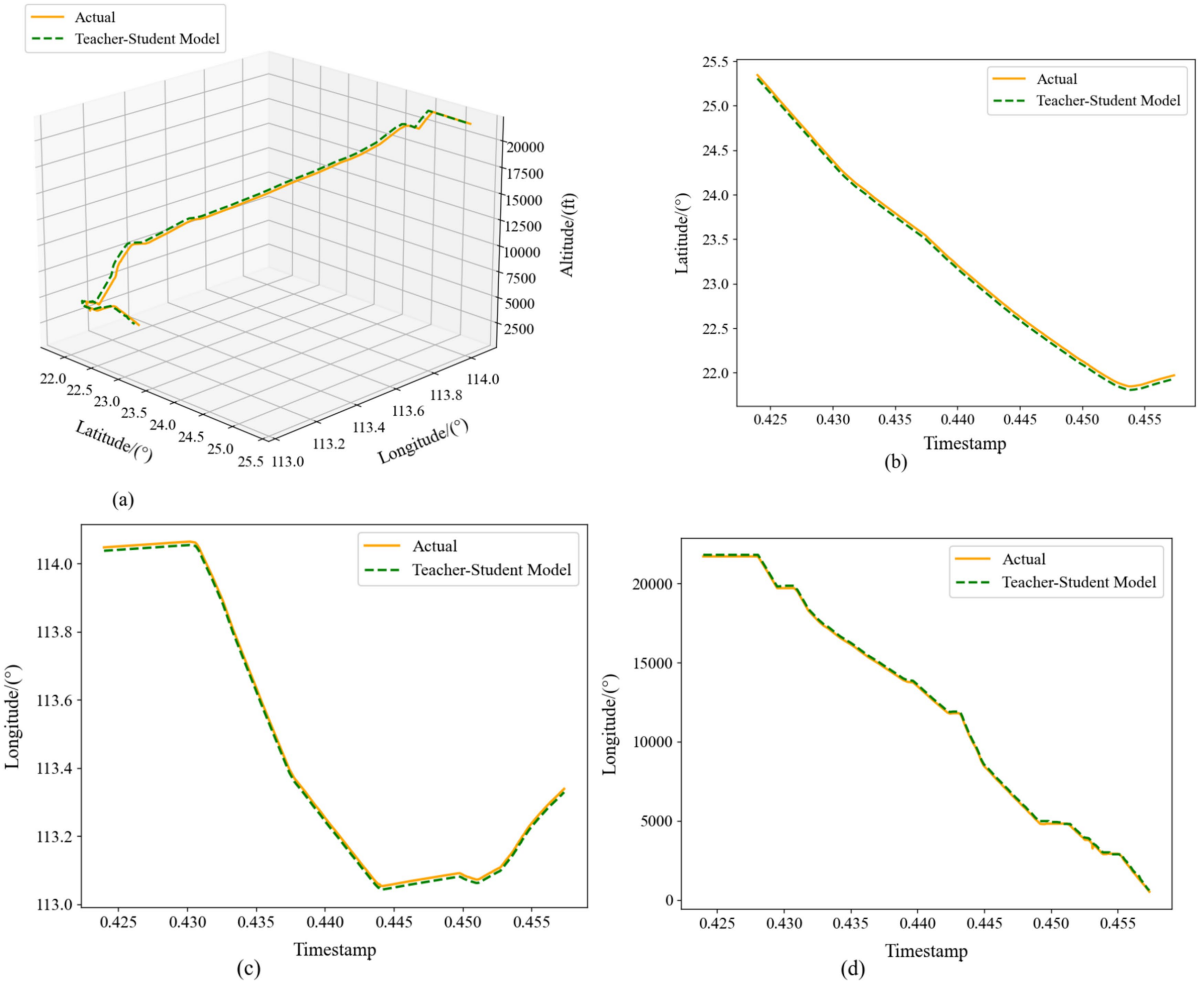
Comparison of single-step prediction of Teacher-Student Model. **(a)** Three-dimensional trajectories. **(b)** Latitude-time plot. **(c)** Longitude-time plot. **(d)** Altitude-time plot.

Figure 12 presents the single-step prediction results of our RCBAM-TCN-LSTM distilled model across longitude, latitude, and altitude, alongside the corresponding 4D trajectory overlay. In the longitude and latitude plots, the predicted curves nearly coincide with the ground truth, with negligible error throughout. The 4D trajectory visualization further confirms that the model faithfully reconstructs each segment of the flight path, closely matching the actual aircraft trajectory.

**Figure 13:**
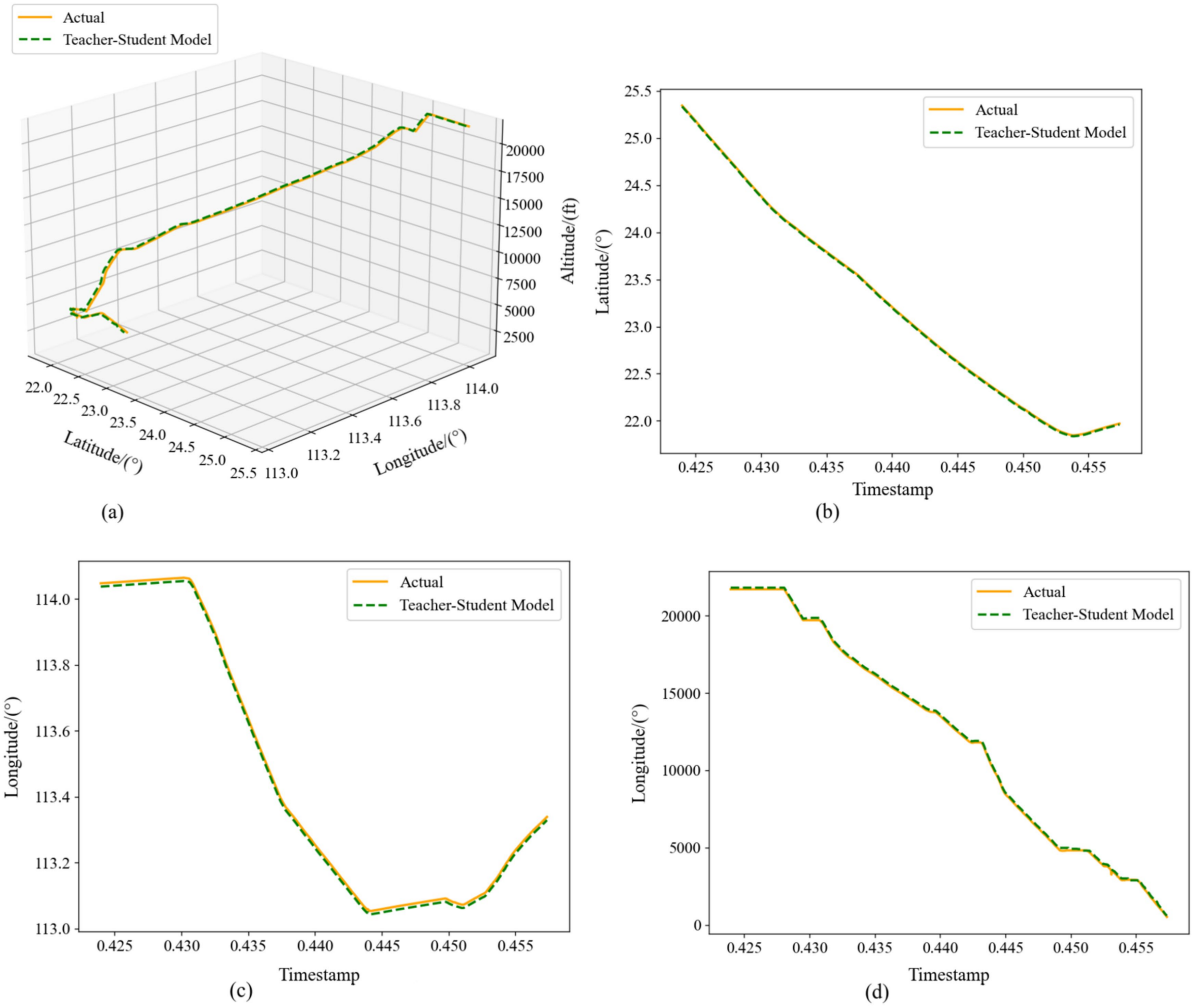
Comparison of multi-step prediction of Teacher-Student Model. **(a)** Three-dimensional trajectories. **(b)** Latitude-time plot. **(c)** Longitude-time plot. **(d)** Altitude-time plot.

Figure 13 illustrates the multi-step predictions in the same four subplots. Even over an extended horizon, the distilled model maintains high fidelity: latitude and longitude deviations shrink further, and altitude predictions more closely track the true profile—especially in steep climbs and descents—than in the single-step case. The 4D trajectory map shows that the predicted path comprehensively covers the real trajectory, exhibiting greater continuity and spatial coherence compared to the original network outputs.

Taken together, Figures 12, 13 demonstrate that our distilled framework effectively fuses the teacher’s fine-grained spatial representations with the student’s long-range temporal memory in single-step forecasts, and further refines accuracy over longer windows through iterative, context-aware learning. These results confirm that combining knowledge distillation with a multi-step prediction strategy yields a robust and precise solution for 4D trajectory forecasting.

### 5.5 Baseline model comparison experiment

To verify the generalization performance of the Teacher-Student Model, this model was compared with existing mainstream trajectory prediction models, including LSTM, GRU, BiLSTM (Zhou et al., 2024), CNN-LSTM (Wang et al., 2025), and TCN-LSTM-attention (Li et al., 2025). All models were evaluated using the same ADS-B dataset. The sliding window size L was set to 60, and the number of filters for LSTM and GRU in each baseline model was set to 64. These models were used to predict the spatial positions of trajectory points 10 steps ahead. For quantitative analysis of the prediction results, MAE, RMSE, MAPE, and *R*^2^ were adopted as error evaluation metrics to measure the accuracy of trajectory prediction.

**Figure 14:**
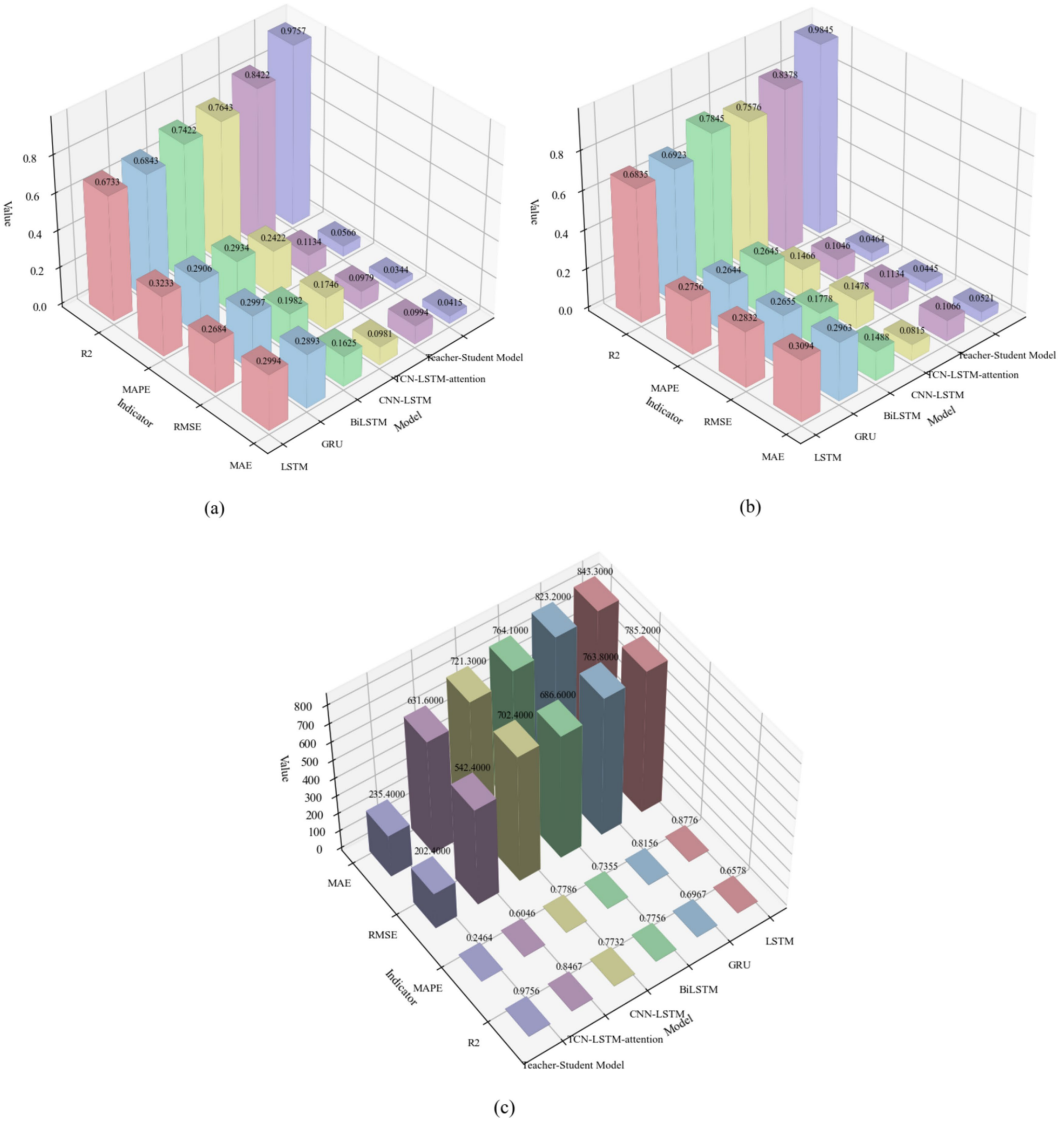
Comparison of model evaluation metrics. **(a)** Longitude. **(b)** Latitude. **(c)** Altitude.

The analysis of the prediction results based on the three geographic dimensions of longitude, latitude, and altitude shows that the Teacher-Student Model (RCBAM-TCN-LSTM Knowledge Distillation Model) significantly outperforms the other baseline models in all the assessment indicators. As can be visualized from the comparison of the model assessment metrics in Table 5, the Teacher-Student Model performs most prominently in terms of the *R*^2^ coefficient of determination, reaching 0.9757 in the longitude prediction, 0.9845 in the latitude prediction, and 0.9756 in the altitude prediction, which is significantly better compared to the traditional LSTM model (0.6733 for latitude, 0.6835 for longitude, and 0.6578 for altitude), respectively. 0.6578) by about 45, 44 and 48%, respectively. The 4D bar chart in Figure 14 clearly shows the performance gap between the models, and the Teacher-Student Model presents the highest bar heights in all the evaluation dimensions, indicating that the knowledge distillation architecture effectively improves the model fitting ability and prediction accuracy.

**Table 5:** Evaluation metrics of different baseline models.

Evaluation metrics	Prediction model	Longitude/°	Latitude/°	Altitude/ft
MAE	LSTM	0.2994	0.3094	843.3
	GRU	0.2893	0.2963	823.2
	BiLSTM	0.1625	0.1488	764.1
	CNN-LSTM	0.0981	0.0815	721.3
	TCN-LSTM-attention	0.0994	0.1066	631.6
	Teacher-Student Model	0.0415	0.0521	235.4
RMSE	LSTM	0.2684	0.2832	785.2
	GRU	0.2997	0.2655	763.8
	BiLSTM	0.1982	0.1778	686.6
	CNN-LSTM	0.1746	0.1478	702.4
	TCN-LSTM-attention	0.0979	0.1134	542.4
	Teacher-Student Model	0.0344	0.0445	202.4
MAPE	LSTM	0.3233	0.2756	0.8776
	GRU	0.2906	0.2644	0.8156
	BiLSTM	0.2934	0.2645	0.7355
	CNN-LSTM	0.2422	0.1466	0.7786
	TCN-LSTM-attention	0.1134	0.1046	0.6046
	Teacher-Student Model	0.0566	0.0464	0.2464
*R* ^2^	LSTM	0.6733	0.6835	0.6578
	GRU	0.6843	0.6923	0.6967
	BiLSTM	0.7422	0.7845	0.7756
	CNN-LSTM	0.7643	0.7576	0.7732
	TCN-LSTM-attention	0.8422	0.8378	0.8467
	Teacher-Student Model	0.9757	0.9845	0.9756

**Figure 15:**
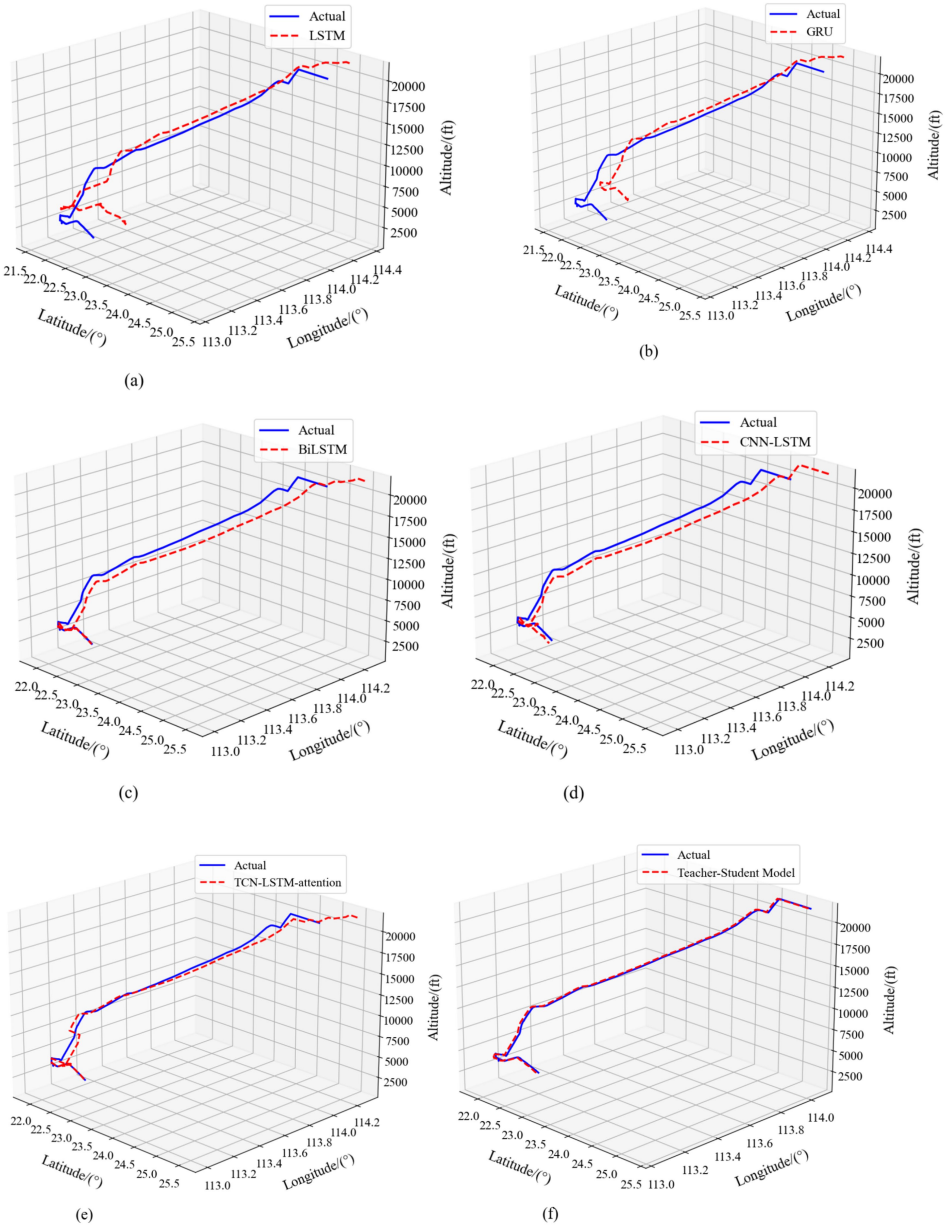
4D comparison of Teacher-Student Model with the baseline model. **(a)** LSTM model. **(b)** GRU model. **(c)** BiLSTM model. **(d)** CNN-LSTM model. **(e)** TCN-LSTM-attention model. **(f)** Teacher-Student Model.

In terms of error control, the Teacher-Student Model exhibits excellent performance. As shown in Figure 14, the model’s MAE index is the lowest value in all three dimensions, with only 0.0415 MAE for longitude prediction, 0.0521 MAE for latitude prediction, and 235.4 MAE for height prediction. Compared with other baseline models, such as the GRU model, which has a MAPE of 0.3233, 0.2756 for the longitudinal, latitudinal, and height dimensions, respectively, 763.8000, the Teacher-Student Model shows significant advantages in longitude and latitude prediction. This error control ability is not only reflected in the numerical indicators, but also intuitively verified in the 4D trajectory comparison visualization results in Figure 15. From Figure 15, it can be clearly observed that compared to baseline models such as LSTM, GRU, BiLSTM, CNN-LSTM, and TCN-LSTM-attention, the predicted trajectory (red dashed line) of Teacher-Student Model has the highest degree of overlap with the real trajectory (blue solid line), and in particular, the key turning points and altitude changes of the trajectory regions show better fitting results.

The teacher–student framework delivers clear structural advantages over any single-architecture model by unifying RCBAM’s spatial attention with TCN-LSTM’s temporal modeling. As shown in Table 5 and Figure 14, in longitude prediction our model achieves an *R*^2^ of 0.9773—an increase of 43% over GRU (0.6843) and 31% over BiLSTM (0.7422). For latitude, *R*^2^ improves by 42% relative to GRU (0.6923) and 25% relative to BiLSTM (0.7845). In altitude prediction the teacher–student model attains an *R*^2^ of 0.9756, outperforming LSTM (0.6578) by 48% and GRU (0.6967) by 40%. Figure 15’s trajectory overlays further corroborate these gains. The LSTM and GRU baselines exhibit substantial deviations on complex segments; BiLSTM and CNN-LSTM reduce but do not eliminate localized errors; and even the TCN-LSTM-attention model—while stronger—fails to match the consistent accuracy of our distilled framework. Notably, height-dimension comparisons in Figure 14c and the vertical trajectory traces in Figure 15 highlight our model’s superior cross-dimensional consistency: with an *R*^2^ of 0.9756 in altitude, it exceeds the TCN-LSTM-attention model’s 0.8467 by over 15%.

The 4D visualizations in Figure 15 confirm that the teacher–student model tracks real flight paths across longitude, latitude, and altitude with remarkable fidelity, aligning perfectly with its leading quantitative performance across all metrics in Figure 14. In summary, by fusing attention mechanisms, temporal convolutions, and long-short-term memory, the RCBAM-TCN-LSTM knowledge-distillation model achieves a comprehensive uplift in geographic coordinate forecasting.

### 5.6 Evaluation of model efficiency

As can be seen from the above Table 6, the RCBAM-TCN-LSTM model after knowledge distillation achieves significant lightweight and acceleration effects while maintaining high prediction accuracy. Compared with the original teacher model, its number of parameters has been reduced by more than 58%, the model file size has been reduced by nearly 59%, the inference delay has been reduced to less than half on the CPU platform, and it can complete a full prediction within 25 ms on the Jetson Nano edge device, fully meeting the requirements of real-time applications. The memory usage has been reduced from 150 MB to only 65 MB, making the model more feasible for deployment in resource-constrained embedded or mobile terminal environments. In contrast, although the pure LSTM performs slightly better in terms of delay, its prediction accuracy is much lower than that of the distilled model; CNN-LSTM is not conducive to edge deployment due to higher delay and memory overhead. In summary, the RCBAM-TCN-LSTM knowledge distillation framework achieves a good balance among accuracy, speed, and resource usage, making it most suitable for real-time or edge-based 4D trajectory prediction scenarios.

**Table 6:** Inference performance and resource usage comparison.

Model	Params (M)	Size (MB)	Inference Latency (CPU ms/Jetson Nano ms)	Memory Usage (MB)
RCBAM (Teacher)	2.42	49.2	18.5/52.4	150
TCN-LSTM (Student)	0.82	19.2	7.2/21.8	60
Teacher-Student Model	1.02	20.4	8.1/24.5	65
LSTM	0.64	14.4	9.0/27.1	55
CNN-LSTM	1.34	25.6	12.8/38.3	80

## 6 Discussion and outlook

The proposed RCBAM-TCN-LSTM knowledge-distillation framework demonstrates strong performance in 4D trajectory prediction by combining the teacher’s spatial attention strengths with the student’s long-range temporal modeling, thus achieving an effective balance between accuracy and efficiency. Compared to both the undistilled teacher and student networks, our distilled model consistently reduces error across multiple metrics while substantially shrinking model size and inference time, enabling real-time prediction on standard CPU and embedded devices and confirming its lightweight, deployable nature.

Nonetheless, two limitations remain. First, our experiments are confined to data from a single airport, leaving the model’s generalizability to other airspaces and operational scenarios untested. Second, under extreme weather conditions or aggressive maneuvers, localized prediction errors tend to increase, indicating that the model’s adaptability to sudden distribution shifts needs further enhancement.

To address these issues, future work will proceed in three directions. We will assemble a large, multi-site, multi-aircraft trajectory dataset and explore domain-adaptation and transfer-learning strategies to improve cross-scenario performance. Next, we plan to integrate meteorological radar and other heterogeneous inputs through cross-modal fusion to bolster robustness in complex weather and airspace conditions. Finally, we will develop an online continual-distillation mechanism so that the edge-deployed student model can continuously update its knowledge as new routes and aircraft types emerge. These enhancements are expected to further strengthen the model’s universality and stability, supporting more reliable intelligent airspace management and autonomous flight decision-making.

## Data Availability

The original contributions presented in the study are included in the article/supplementary material, further inquiries can be directed to the corresponding author.

## Footnotes

^1^ http://www.variflight.com/

## References

[ref1] Bai S. Kolter J. Koltun V. (2018). An empirical evaluation of generic convolutional and recurrent networks for sequence modeling. arXiv preprint 3, 1–14.

[ref2] Chang J. Liu C. Zheng Q. Li Y. (2020). Summary and outlook of 4D track prediction methods. J. Mech. Eng. Autom. Control Syst. 1, 46–55. doi: 10.21595/jmeacs.2020.21553

[ref3] Chuan Y. Jing D. Juan Y. Zhong H. F. (2023). The high-speed aircraft trajectory prediction method based on deep learning network. J. Phys. Conf. Ser. 2489:012023. doi: 10.1088/1742-6596/2489/1/012023

[ref4] Dong Z. Fan B. Li F. Xu X. Sun H. Cao W. (2023). TCN-informer-based flight trajectory prediction for aircraft in the approach phase. Sustainability 15:16344. doi: 10.3390/su152316344

[ref5] Gao Z. Zhang D. Yi W. (2025). Projectile trajectory and launch point prediction based on CORR-CNN-BiLSTM-attention model. Expert Syst. Appl. 275:127045. doi: 10.1016/j.eswa.2025.127045

[ref6] Gou J. Chen Y. Yu B. Liu J. du L. Wan S.. (2024). Reciprocal teacher-student learning via forward and feedback knowledge distillation. IEEE Trans. Multimed. 26, 7901–7916. doi: 10.1109/TMM.2024.3372833

[ref7] Guo D. Zhang Z. Yang B. Zhang J. Yang H. Lin Y. (2024). Integrating spoken instructions into flight trajectory prediction to optimize automation in air traffic control. Nat. Commun. 15:9662. doi: 10.1038/s41467-024-54069-5, PMID: 39511176 PMC11543814

[ref8] Han P. Wang W. Shi Q. Yue J. (2021). A combined online-learning model with K-means clustering and GRU neural networks for trajectory prediction. Ad Hoc Netw. 117:102476. doi: 10.1016/j.adhoc.2021.102476

[ref9] Han P. Zhang Q. Shi Q. Zhang Z. (2023). Terminal area 4D trajectory prediction based on DBSCAN-GRU algorithm. Signal Process. 39, 439–449.

[ref10] Hao S. Cheng S. Zhang Y. (2018). A multi-aircraft conflict detection and resolution method for 4-dimensional trajectory-based. Chin. J. Aeronaut. 31, 1579–1593. doi: 10.1016/j.cja.2018.04.017

[ref11] Huang J. Ding W. (2022). Aircraft trajectory prediction based on Bayesian optimized temporal convolutional network-bidirectional gated recurrent unit hybrid neural network. Int. J. Aerospace Eng. 2022, 1–19.

[ref12] Lei D. Xu M. Wang S. (2025). A deep multimodal network for multi-task trajectory prediction[J]. Inf. Fusion 113:102597. doi: 10.1016/j.inffus.2024.102597

[ref13] Li Y. Fang Y. Long T. (2025). Noise robust aircraft trajectory prediction via autoregressive transformers with hybrid positional encoding. Sci. Rep. 15:11370. doi: 10.1038/s41598-025-96512-7, PMID: 40175447 PMC11965515

[ref14] Li L. Shi K. Mao Q.. (2025). TBM tunneling speed prediction based on TCN-LSTM-attention model. Yangtze River, 1–18. Available at: https://link.cnki.net/urlid/42.1202.TV20250509.2140.008

[ref15] Liu Y. Hansen M. (2018). Predicting aircraft trajectories: a deep generative convolutional recurrent neural networks approach. ArXiv.

[ref16] Lu T. Liu B. Li C. (2024). Aircraft trajectory prediction in terminal area based on attention Seq2Seq model. Sci. Technol. Eng. 24, 3882–3895.

[ref17] Ma L. Meng X. Wu Z. (2024). Data-driven 4D trajectory prediction model using attention-TCN-GRU. Aerospace 11:313. doi: 10.3390/aerospace11040313

[ref18] Peng Y. Hu M. Zhang Y. (2005). Dynamic trajectory speculation method. J. Transp. Eng. 5, 61–65.

[ref19] Ramasamy S. Sabatini R. Gardi A. (2014). Next generation flight management system for real-time trajectory based operations. Appl. Mech. Mater. 629, 344–349. doi: 10.4028/www.scientific.net/AMM.629.344

[ref20] Sahadevan D. Ponnusamy P. Gopi V. Nelli M. K. (2022). Ground-based 4d trajectory prediction using bi-directional LSTM networks. Appl. Intell. 52, 16417–16434. doi: 10.1007/s10489-022-03309-6

[ref21] Shafienya H. Regan A. (2022). 4D flight trajectory prediction using a hybrid deep learning prediction method based on ADS-B technology: a case study of Hartsfield-Jackson Atlanta international airport (ATL). Transp. Res. C 144:103878. doi: 10.1016/j.trc.2022.103878

[ref22] Shen T. Wang J. Zhang X. (2025). Knowledge distillation via adaptive meta-learning for graph neural network. Inf. Sci. 689:121505. doi: 10.1016/j.ins.2024.121505

[ref23] Shi Z. Xu M. Pan Q. Yan B. Zhang H. (2018). LSTM-based flight trajectory prediction. In 2018 international joint conference on neural networks (IJCNN). Rio de Janeiro, Brazil: IEEE, 1–8.

[ref24] Shi Q. Yue J. Han P. (2019). Short-term flight trajectory prediction based on LSTM-ARIMA model. Signal Process. 35, 2000–2009.

[ref25] Shi Q. Zhao W. Han P. (2024). GTA-Seq2Seq multi-step 4D trajectory prediction model based on spatio-temporal feature extraction. J. Beijing Univ. Aeronaut. Astronaut., 1–16. Available at: https://link.cnki.net/doi/10.13700/j.bh.1001-5965.2024.0679

[ref26] Tang X. Li T. Chen Q. (2020). Short-term 4D track prediction based on interactive multi-modeling. J. Wuhan Univ. Technol. 44, 39–45.

[ref27] Vaugeois M. (2018). The 13th air navigation conference. Proceedings of the 13th air navigation conference Montreal. International Civil Aviation Organization, 222–239.

[ref28] Wang Z. Fang X. Zhang W. Wang L. Wang K. Chen C. (2025). Dynamic intelligent prediction approach for landslide displacement based on biological growth models and CNN-LSTM. J. Mt. Sci. 22, 71–88. doi: 10.1007/s11629-024-9113-y

[ref29] Wu Z. Tian S. Ma L. (2019). A 4D trajectory prediction model based on the BP neural network. J. Intell. Syst. 29, 1545–1557. doi: 10.1515/jisys-2019-0077

[ref30] Wu X. Yang H. Chen H. Hu Q. Hu H. (2022). Long-term 4D trajectory prediction using generative adversarial networks. Transp. Res. Part C Emerg. Technol. 136:103554. doi: 10.1016/j.trc.2022.103554

[ref31] Xie L. Cen X. Lu H. Yin G. Yin M. (2024). A hierarchical feature-logit-based knowledge distillation scheme for internal defect detection of magnetic tiles. Adv. Eng. Inform. 61:102526. doi: 10.1016/j.aei.2024.102526

[ref32] Zeng W. Chu X. Xu Z. Liu Y. Quan Z. (2022). Aircraft 4D trajectory prediction in civil aviation: a review. Aerospace 9:91. doi: 10.3390/aerospace9020091

[ref33] Zhang H. Liu Z. (2025). Four-dimensional aircraft trajectory prediction with a generative deep learning and clustering approach. J. Aerospace Inf. Syst. 22, 90–102. doi: 10.2514/1.I011454

[ref34] Zhang Y. Zhou G. Xie Z. Ma J. Huang J. X. (2025). A diversity-enhanced knowledge distillation model for practical math word problem solving. Inf. Process. Manag. 62:104059. doi: 10.1016/j.ipm.2025.104059

[ref35] Zhao Z. Zeng W. Quan Z. Chen M. (2019). Aircraft trajectory prediction using deep long short-term memory networks. in: 19th COTA international conference of transportation, pp. 1–14.

[ref36] Zhou Z. Hou H. Sun L. (2024). Research on solar heating load prediction based on CNN and BiLSTM neural network models. Acta Energiae Solaris Sinica 45, 415–422.

[ref37] Zhou X. Wu J. Liang W. Wang K. I. K. Yan Z. Yang L. T.. (2024). Reconstructed graph neural network with knowledge distillation for lightweight anomaly detection. IEEE Trans. Neural Netw. Learn. Syst. 35, 11817–11828. doi: 10.1109/TNNLS.2024.3389714, PMID: 38687671

